# K_0.12_Na_0.54_Ag_0.34_Nb_4_O_9_AsO_4_

**DOI:** 10.1107/S1600536811000055

**Published:** 2011-01-12

**Authors:** Saïda Fatma Chérif, Mohamed Faouzi Zid, Ahmed Driss

**Affiliations:** a Laboratoire de Matériaux et Cristallochimie, Faculté des Sciences de Tunis, Université de Tunis El Manar, 2092 Manar II Tunis, Tunisia

## Abstract

Potassium sodium silver tetra­niobium nona­oxide arsenate, K_0.12_Na_0.54_Ag_0.34_Nb_4_AsO_13_, synthesized by solid-state reaction at 1123 K, adopts a three-dimensional framework delimiting tunnels running along [001] in which occupationally disordered sodium, silver, and potassium ions are located. Of the 11 atoms in the asymmetric unit (two Nb, one As, one Ag, one K, one Na and fiveO), nine are located on special positions: one Nb and the K, Ag, Na and two O atoms are situated on mirror planes, the other Nb is on a twofold rotation axis, and the As atom and one O atom are on sites of *m*2*m* symmetry.

## Related literature

For physical properties, see: Piffard *et al.* (1985 ▶); Lachgar *et al.* (1986 ▶); Harrison *et al.* (1994 ▶); Ledain *et al.* (1997 ▶); Hizaoui *et al.* (1999 ▶); Zid *et al.* (2003 ▶); Hajji *et al.* (2004 ▶). For synthetic details, see: Zid *et al.* (1988 ▶, 1989 ▶); Haddad *et al.* (1988 ▶); Ben Amor & Zid (2006 ▶). For structural relationships, see: Ben Amor *et al.* (2008 ▶); Bestaoui *et al.* (1998 ▶); Haddad *et al.* (1988 ▶). For bond-valence sums, see: Brown & Altermatt (1985 ▶).

## Experimental

### 

#### Crystal data

K_0.12_Na_0.54_Ag_0.34_Nb_4_AsO_13_
*M* *_r_* = 708.82
Orthorhombic, 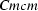

*a* = 10.412 (2) Å
*b* = 10.452 (2) Å
*c* = 10.009 (4) Å
*V* = 1089.2 (5) Å^3^
*Z* = 4
Mo *K*α radiation
μ = 7.86 mm^−1^
*T* = 298 K
0.22 × 0.16 × 0.10 mm

#### Data collection

Enraf–Nonius CAD-4 diffractometer
Absorption correction: ψ scan (North *et al.*, 1968 ▶) *T*_min_ = 0.239, *T*_max_ = 0.452
1550 measured reflections
667 independent reflections
642 reflections with *I* > 2σ(*I*)
*R*_int_ = 0.014
2 standard reflections every 120 min  intensity decay: 1.1%

#### Refinement

*R*[*F* ^2^ > 2σ(*F* ^2^)] = 0.013
*wR*(*F* ^2^) = 0.035
*S* = 1.14
667 reflections
72 parameters
1 restraint
Δρ_max_ = 0.42 e Å^−3^
Δρ_min_ = −0.57 e Å^−3^

### 

Data collection: *CAD-4 EXPRESS* (Duisenberg, 1992 ▶; Macíček & Yordanov, 1992 ▶); cell refinement: *CAD-4 EXPRESS*; data reduction: *XCAD4* (Harms & Wocadlo, 1995 ▶); program(s) used to solve structure: *SHELXS97* (Sheldrick, 2008 ▶); program(s) used to refine structure: *SHELXL97* (Sheldrick, 2008 ▶); molecular graphics: *DIAMOND* (Brandenburg, 1998 ▶); software used to prepare material for publication: *WinGX* (Farrugia, 1999 ▶).

## Supplementary Material

Crystal structure: contains datablocks I, global. DOI: 10.1107/S1600536811000055/mg2111sup1.cif

Structure factors: contains datablocks I. DOI: 10.1107/S1600536811000055/mg2111Isup2.hkl

Additional supplementary materials:  crystallographic information; 3D view; checkCIF report

## Figures and Tables

**Fig. 1.**
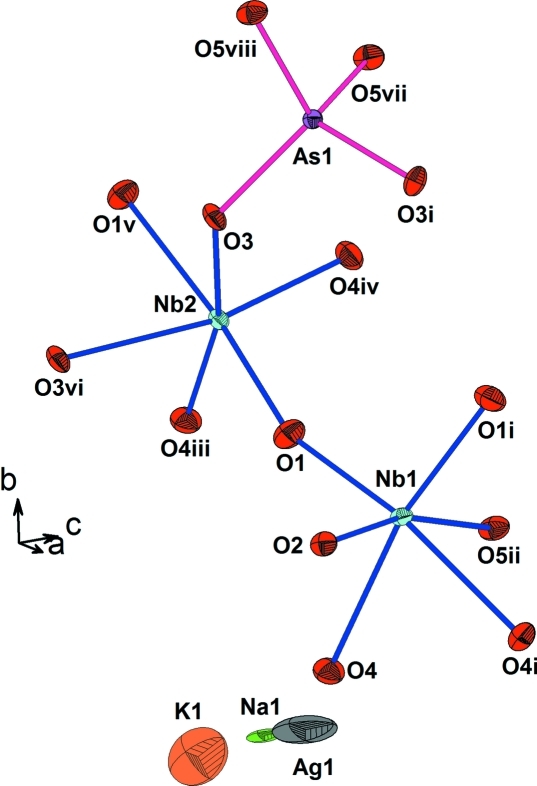
Unité asymétrique linéaire dans K0.12Na0.54Ag0.34Nb4AsO13. Les éllipsoïdes ont été définis avec 50% de probabilité. [Code de symétrie: (i) x, y, -z + 1/2; (ii) x - 1/2, y - 1/2, z; (iii) -x + 1/2, -y + 1/2, -z; (iv) -x + 1/2, y + 1/2, z; (v) x, -y + 1, -z; (vi) -x, -y + 1, -z; (vii) -x + 1, y, z; (viii) x - 1, y, z].

**Fig. 2.**
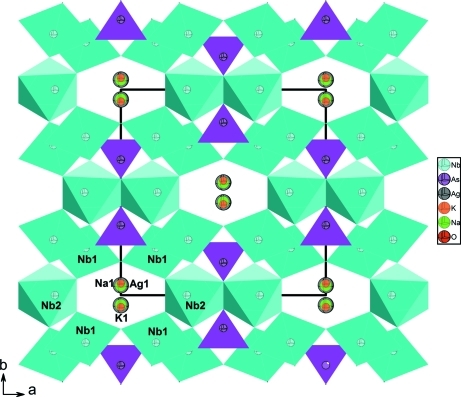
Projection de la structure de K0.12Na0.54Ag0.34Nb4AsO13 selon c, montrant les canaux où se situent les cations.

**Fig. 3.**
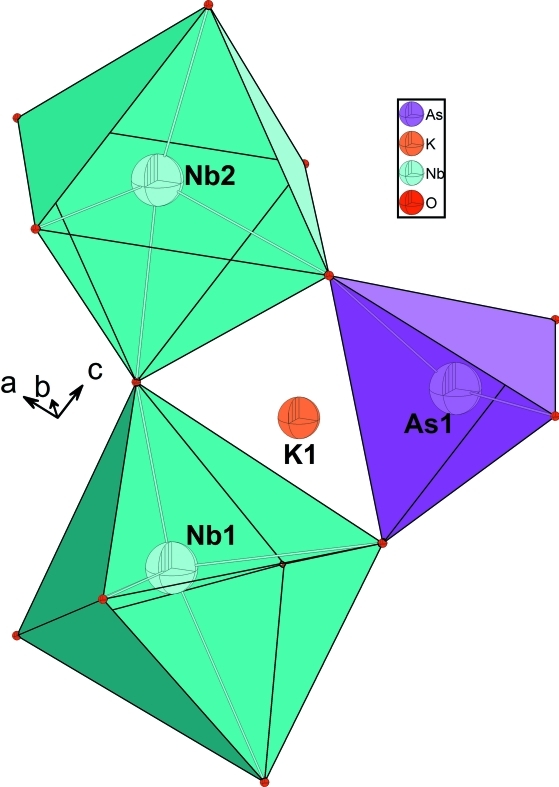
Unité asymétrique cyclique dans KNb4AsO13.

## supplementary crystallographic information

### Comment

*L*'élaboration de nouveaux matériaux pouvant être potentiellement des conducteurs ioniques (Piffard *et al.*, 1985), être utilisé comme tamis moléculaires ou bien en catalyse hétérogène (Ledain *et al.*, 1997), a conduit à s'intéresser aux composés à charpentes ouvertes mixtes constituées d'octaèdres *M*O_6_ [*M* = Sb(Lachgar *et al.*, 1986), Nb (Hizaoui *et al.*, 1999), Mo (Hajji *et al.*, 2004)] et de tétraèdres XO_4_ [*X* = P (Zid *et al.*, 2003), As (Harrison *et al.*, 1994)]. C'est dans ce cadre que nous avons entrepris l'étude des systèmes A—Nb—As—O (A = alcalin, argent) dans lesquels nous avons précédemment caractérisé les phases suivantes: K_3_NbAs_2_O_9_ (Zid *et al.*, 1989), Ag_3_Nb_3_As_2_O_14_ (Ben Amor & Zid, 2006), K_2_Nb_2_As_2_O_11_ (Zid *et al.*, 1988) et KNb_4_AsO_13_ (Haddad *et al.*, 1988). Nous reportons dans ce travail le mode de préparation et la structure déterminée par diffraction des rayons-*X* sur monocristal d'un oxyde mixte de formulation K_0.12_Na_0.54_Ag_0.34_Nb_4_AsO_13_. *L*'unité *asym*étrique dans le composé K_0.12_Na_0.54_Ag_0.34_Nb_4_AsO_13_ est construite à partir d'un tétraèdre As(1)O_4_ et deux octaèdres NbO_6_ reliés par mize en commun de sommets (Fig. 1). Ces unités se connectent entre elles pour former des chaînes infinies (Nb(1)_2_AsO_14_)_n_ de type (Nb(1)O_6_—Nb(1)O_6_—As(1)O_4_) disposées selon la direction [100]. La jonction entre ces chaînes est assurée par mize en commun de sommets avec des paires d'octaèdres Nb(2)O_6_ partageant des arêtes. Il en résulte une charpente tridimensionnelle possédant des canaux, à section hexagonale, où se situent les cations Ag^+^, Na^+^ et K^+^ (Fig. 2). Le calcul des différentes valences des liaisons utilisant la formule empirique de Brown (Brown & Altermatt, 1985) vérifie bien les valeurs de charges des ions: Nb1 (5,08), Nb2 (5,01), As1 (4,95), Ag1 (0,74), K1 (0,87) e t Na1 (0,66) attendues dans la phase étudiée. La comparaison de la structure du composé étudié avec celles des travaux antérieurs montre qu'elle est apparentée à la famille suivante: K_0.2_Ag_0.8_Nb_4_AsO_13_ (Ben Amor *et al.*, 2008), KNb_4_AsO_13_ (Haddad *et al.*, 1988) et NaNb_4_AsO_13_ (Bestaoui *et al.*, 1998). Elles ont la même charpente anionique. Cependant, une différence s'observe au niveau de l'unité *asym*étrique. En effet, dans les composés K_0.2_Ag_0.8_Nb_4_AsO_13_, NaNb_4_AsO_13_ et K_0.12_Na_0.54_Ag_0.34_Nb_4_AsO_13_, l'unité *asym*étrique est linéaire (Fig. 1), tandis qu'elle est cyclique dans le composé KNb_4_AsO_13_ (Fig. 3). De plus, l'examen des facteurs géométriques dans les quatre composés montre que l'environnement et la coordination des cations monovalents sont différents. En effet, dans la structure du composé KNb_4_AsO_13_, le cation K^+^ occupe totalement son site et il est entouré par sept atomes d'oxygène, alors que dans la phase étudiée K_0.12_Na_0.54_Ag_0.34_Nb_4_AsO_13_ et celle de NaNb_4_AsO_13_, chaque cation Na^+^ occupe partiellement son site et il est entouré seulement par cinq atomes d'oxygène. Dans la structure de K_0.2_Ag_0.8_Nb_4_AsO_13_, les cations Ag^+^ et K^+^ occupent partiellement leurs sites. Chaque cation K^+^ est entouré par cinq atomes d'oxygène tandis que dans notre composé, il est environné par six. Si on se limite à la première sphère de coordination, les cations Ag^+^, dans les deux derniers oxydes, sont entourés par trois atomes d'oxygène. Afin d'utiliser ces données structurales et les relier aux propriétés physico-chimiques, en particulier de conduction ionique, et dès l'obtention d'une phase pure du composé étudié de formulation K_0.12_Na_0.54_Ag_0.34_Nb_4_AsO_13_, des mesures électriques moyennant un pont d'impédance complexe de type HP4192A seront réalisées.

### Experimental

Les cristaux relatifs à la phase K_0.12_Na_0.54_Ag_0.34_Nb_4_AsO_13_ ont été obtenus à partir des réactifs: Nb_2_O_5_ (Fluka, 72520), NH_4_H_2_AsO_4_ (préparé au laboratoire, ASTM 01–775), AgNO_3_ (Fluka, 69658), K_2_CO_3_ (Fluka, 60109) e t Na_2_CO_3_ (Prolabo, 74136) pris dans les proportions Na:K:Ag:Nb:As = 1:1:1:3:2. Le mélange, finement broyé, a été mis dans un creuset en porcelaine, placé dans un four puis préchauffé à l'air à 523 K pendant 24 heures en vue d'éliminer les composés volatils. Il est ensuite porté à une température proche de sa fusion, 1123 K. Le mélange est abandonné à cette température pendant une semaine pour favoriser la germination et la croissance des cristaux. Le résidu final a subi en premier lieu un refroidissement lent (5 K h^-1^) jusqu'à 1023 K puis un second rapide (50 K h^-1^) jusqu'à la température ambiante. Des cristaux incolores, de taille suffisante pour les mesures des intensités, ont été séparés du flux par l'eau bouillante. Une analyse qualitative au M.E.B. de type FEI Quanta 200 d'un cristal de la phase, confirme la présence des différents éléments chimiques attendus notamment: Na, K, Ag, Nb, As et l'oxygène.

### Refinement

Dans l'affinement final et pour des raisons de neutralité électrique les taux d'occupation des cations Na^+^, K^+^ et Ag^+^ ont été menés en utilisant la condition SUMP autorisée par le programme *SHELX. L*'affinement de tous les paramètres variables conduit à des ellipsoïdes bien définies. Les densités d'électrons maximum et minimum restants dans la Fourier-différence sont situées respectivements à 0,51 Å de O2 et à 0,73 Å de A s1.

### Crystal data

**Table:**

K_0.12_Na_0.54_Ag_0.34_Nb_4_AsO_13_	*F*(000) = 1302
*M**_r_* = 708.82	*D*_x_ = 4.322 Mg m^−^^3^
Orthorhombic, *C**m**c**m*	Mo *K*α radiation, λ = 0.71073 Å
Hall symbol: -C 2c 2	Cell parameters from 25 reflections
*a* = 10.412 (2) Å	θ = 10–15°
*b* = 10.452 (2) Å	µ = 7.86 mm^−^^1^
*c* = 10.009 (4) Å	*T* = 298 K
*V* = 1089.2 (5) Å^3^	Prism, colourless
*Z* = 4	0.22 × 0.16 × 0.10 mm

### Data collection

**Table:**

Enraf–Nonius CAD-4 diffractometer	642 reflections with *I* > 2σ(*I*)
Radiation source: fine-focus sealed tube	*R*_int_ = 0.014
graphite	θ_max_ = 26.9°, θ_min_ = 2.8°
ω/2θ scans	*h* = −13→13
Absorption correction: ψ scan (North *et al.*, 1968)	*k* = −1→13
*T*_min_ = 0.239, *T*_max_ = 0.452	*l* = −1→12
1550 measured reflections	2 standard reflections every 120 min
667 independent reflections	intensity decay: 1.1%

### Refinement

**Table:**

Refinement on *F*^2^	Primary atom site location: structure-invariant direct methods
Least-squares matrix: full	Secondary atom site location: difference Fourier map
*R*[*F*^2^ > 2σ(*F*^2^)] = 0.013	*w* = 1/[σ^2^(*F*_o_^2^) + (0.0164*P*)^2^ + 2.8925*P*] where *P* = (*F*_o_^2^ + 2*F*_c_^2^)/3
*wR*(*F*^2^) = 0.035	(Δ/σ)_max_ = 0.001
*S* = 1.14	Δρ_max_ = 0.42 e Å^−^^3^
667 reflections	Δρ_min_ = −0.57 e Å^−^^3^
72 parameters	Extinction correction: *SHELXL*, Fc^*^=kFc[1+0.001xFc^2^λ^3^/sin(2θ)]^-1/4^
1 restraint	Extinction coefficient: 0.00226 (10)

### Special details

**Table:**

Geometry. All e.s.d.'s (except the e.s.d. in the dihedral angle between two l.s. planes) are estimated using the full covariance matrix. The cell e.s.d.'s are taken into account individually in the estimation of e.s.d.'s in distances, angles and torsion angles; correlations between e.s.d.'s in cell parameters are only used when they are defined by crystal symmetry. An approximate (isotropic) treatment of cell e.s.d.'s is used for estimating e.s.d.'s involving l.s. planes.
Refinement. Refinement of *F*^2^ against ALL reflections. The weighted *R*-factor *wR* and goodness of fit *S* are based on *F*^2^, conventional *R*-factors *R* are based on *F*, with *F* set to zero for negative *F*^2^. The threshold expression of *F*^2^ > σ(*F*^2^) is used only for calculating *R*-factors(gt) *etc*. and is not relevant to the choice of reflections for refinement. *R*-factors based on *F*^2^ are statistically about twice as large as those based on *F*, and *R*- factors based on ALL data will be even larger.

### Fractional atomic coordinates and isotropic or equivalent isotropic displacement parameters (Å^2^)

**Table:**

	*x*	*y*	*z*	*U*_iso_*/*U*_eq_	Occ. (<1)
Nb1	0.17145 (3)	0.23034 (3)	0.2500	0.00637 (11)	
Nb2	0.17813 (3)	0.5000	0.0000	0.00638 (11)	
As1	0.0000	0.65963 (5)	0.2500	0.00553 (13)	
Ag1	0.0000	−0.0486 (8)	0.2165 (6)	0.044 (13)	0.173 (4)
K1	0.0000	−0.057 (3)	0.067 (9)	0.047 (13)	0.059 (13)
Na1	0.0000	−0.0472 (18)	0.160 (3)	0.012 (12)	0.268 (13)
O1	0.15106 (17)	0.34333 (17)	0.11032 (18)	0.0131 (4)	
O2	0.0000	0.1632 (3)	0.2500	0.0090 (7)	
O3	0.0000	0.5664 (2)	0.1105 (2)	0.0095 (5)	
O4	0.21550 (16)	0.07889 (17)	0.11618 (17)	0.0127 (4)	
O5	0.8731 (2)	0.7580 (2)	0.2500	0.0110 (5)	

### Atomic displacement parameters (Å^2^)

**Table:**

	*U*^11^	*U*^22^	*U*^33^	*U*^12^	*U*^13^	*U*^23^
Nb1	0.00591 (16)	0.00475 (17)	0.00844 (16)	0.00075 (10)	0.000	0.000
Nb2	0.00670 (16)	0.00656 (17)	0.00588 (16)	0.000	0.000	−0.00138 (10)
As1	0.0053 (2)	0.0051 (2)	0.0062 (2)	0.000	0.000	0.000
Ag1	0.054 (2)	0.0075 (11)	0.07 (4)	0.000	0.000	0.001 (3)
K1	0.034 (11)	0.048 (14)	0.06 (4)	0.000	0.000	0.007 (18)
Na1	0.012 (4)	0.002 (4)	0.02 (4)	0.000	0.000	0.001 (7)
O1	0.0133 (8)	0.0111 (8)	0.0149 (9)	0.0001 (7)	0.0002 (7)	0.0043 (7)
O2	0.0056 (15)	0.0086 (16)	0.0128 (17)	0.000	0.000	0.000
O3	0.0092 (10)	0.0117 (11)	0.0077 (11)	0.000	0.000	−0.0043 (10)
O4	0.0143 (8)	0.0116 (8)	0.0121 (8)	0.0022 (7)	0.0021 (7)	−0.0026 (7)
O5	0.0068 (11)	0.0090 (11)	0.0173 (13)	0.0010 (9)	0.000	0.000

### Geometric parameters (Å, °)

**Table:**

Nb1—O1^i^	1.8424 (18)	As1—O3^i^	1.703 (2)
Nb1—O1	1.8424 (18)	Ag1—O2	2.239 (9)
Nb1—O2	1.9180 (14)	Ag1—O5^ix^	2.439 (7)
Nb1—O5^ii^	2.119 (3)	Ag1—O5^x^	2.439 (7)
Nb1—O4	2.1236 (18)	K1—O4	2.70 (3)
Nb1—O4^i^	2.1236 (18)	K1—O4^xi^	2.70 (3)
Nb2—O4^iii^	1.8052 (17)	K1—O4^xii^	2.91 (6)
Nb2—O4^iv^	1.8052 (17)	K1—O4^xiii^	2.91 (6)
Nb2—O1	1.9950 (17)	K1—O2	2.94 (8)
Nb2—O1^v^	1.9950 (17)	K1—O5^ix^	2.97 (5)
Nb2—O3^vi^	2.2681 (14)	Na1—O2	2.37 (2)
Nb2—O3	2.2681 (14)	Na1—O5^ix^	2.588 (18)
As1—O5^vii^	1.674 (2)	Na1—O5^x^	2.588 (18)
As1—O5^viii^	1.674 (2)	Na1—O4	2.640 (11)
As1—O3	1.703 (2)	Na1—O4^xi^	2.640 (11)
			
O1^i^—Nb1—O1	98.72 (12)	O4^iii^—Nb2—O1^v^	93.92 (8)
O1^i^—Nb1—O2	97.31 (8)	O4^iv^—Nb2—O1^v^	96.03 (8)
O1—Nb1—O2	97.31 (8)	O1—Nb2—O1^v^	163.76 (10)
O1^i^—Nb1—O5^ii^	91.53 (7)	O4^iii^—Nb2—O3^vi^	162.84 (8)
O1—Nb1—O5^ii^	91.53 (7)	O4^iv^—Nb2—O3^vi^	92.74 (7)
O2—Nb1—O5^ii^	166.39 (12)	O1—Nb2—O3^vi^	84.44 (8)
O1^i^—Nb1—O4	168.89 (8)	O1^v^—Nb2—O3^vi^	82.29 (8)
O1—Nb1—O4	91.38 (8)	O4^iii^—Nb2—O3	92.74 (7)
O2—Nb1—O4	85.90 (9)	O4^iv^—Nb2—O3	162.84 (8)
O5^ii^—Nb1—O4	83.55 (7)	O1—Nb2—O3	82.29 (8)
O1^i^—Nb1—O4^i^	91.38 (8)	O1^v^—Nb2—O3	84.44 (8)
O1—Nb1—O4^i^	168.89 (8)	O3^vi^—Nb2—O3	70.28 (10)
O2—Nb1—O4^i^	85.90 (9)	O5^vii^—As1—O5^viii^	104.25 (17)
O5^ii^—Nb1—O4^i^	83.55 (7)	O5^vii^—As1—O3	110.57 (6)
O4—Nb1—O4^i^	78.20 (10)	O5^viii^—As1—O3	110.57 (6)
O4^iii^—Nb2—O4^iv^	104.31 (11)	O5^vii^—As1—O3^i^	110.57 (6)
O4^iii^—Nb2—O1	96.03 (8)	O5^viii^—As1—O3^i^	110.57 (6)
O4^iv^—Nb2—O1	93.92 (8)	O3—As1—O3^i^	110.19 (17)

Symmetry codes: (i) *x*, *y*, −*z*+1/2; (ii) *x*−1/2, *y*−1/2, *z*; (iii) −*x*+1/2, *y*+1/2, *z*; (iv) −*x*+1/2, −*y*+1/2, −*z*; (v) *x*, −*y*+1, −*z*; (vi) −*x*, −*y*+1, −*z*; (vii) −*x*+1, *y*, *z*; (viii) *x*−1, *y*, *z*; (ix) *x*−1, *y*−1, *z*; (x) −*x*+1, *y*−1, *z*; (xi) −*x*, *y*, *z*; (xii) −*x*, −*y*, −*z*; (xiii) *x*, −*y*, −*z*.
